# TNM-O: ontology support for staging of malignant tumours

**DOI:** 10.1186/s13326-016-0106-9

**Published:** 2016-11-14

**Authors:** Martin Boeker, Fábio França, Peter Bronsert, Stefan Schulz

**Affiliations:** 1 Institute for Medical Biometry and Statistics, Medical Center – University of Freiburg, Faculty of Medicine, Stefan-Meier-Str. 26, Freiburg i. Br., 79104 Germany; 2 Department of Informatics, University of Minho, Campus de Gualtar, Braga, 4710-057 Portugal; 3 Tumorbank Comprehensive Cancer Center Freiburg and Center for Surgical Pathology, Medical Center – University of Freiburg, Faculty of Medicine, Breisacher Straße 115a, Freiburg i. Br., 79106 Germany; 4 Institute of Medical Computer Sciences, Statistics and Documentation, Medical University of Graz, Auenbruggerplatz 2, Graz, 8036 Austria

**Keywords:** TNM classification, Tumour classification, Tumour staging, Anatomical extent, TNM ontology, Description logic

## Abstract

**Background:**

Objectives of this work are to (1) present an ontological framework for the TNM classification system, (2) exemplify this framework by an ontology for colon and rectum tumours, and (3) evaluate this ontology by assigning TNM classes to real world pathology data.

**Methods:**

The TNM ontology uses the Foundational Model of Anatomy for anatomical entities and BioTopLite 2 as a domain top-level ontology. General rules for the TNM classification system and the specific TNM classification for colorectal tumours were axiomatised in description logic. Case-based information was collected from tumour documentation practice in the Comprehensive Cancer Centre of a large university hospital. Based on the ontology, a module was developed that classifies pathology data.

**Results:**

TNM was represented as an information artefact, which consists of single representational units. Corresponding to every representational unit, tumours and tumour aggregates were defined. Tumour aggregates consist of the primary tumour and, if existing, of infiltrated regional lymph nodes and distant metastases. TNM codes depend on the location and certain qualities of the primary tumour (T), the infiltrated regional lymph nodes (N) and the existence of distant metastases (M). Tumour data from clinical and pathological documentation were successfully classified with the ontology.

**Conclusion:**

A first version of the TNM Ontology represents the TNM system for the description of the anatomical extent of malignant tumours. The present work demonstrates its representational power and completeness as well as its applicability for classification of instance data.

## Background

Clinical and pathological staging of malignant tumours is one of the most important procedures in the diagnosis of cancer for prognosis assessment and treatment planning. The staging procedure compiles several clinical and pathological parameters such as the location and the size of the *primary* tumour, the location and the number of the infiltrated *regional* lymph nodes, and the existence of distant metastases.

A prerequisite for an evidence-based cancer treatment is a correct and unambiguous cancer diagnosis. Interdisciplinary expert groups, e.g. from clinical medicine, imaging, and pathology, have been working in close cooperation to establish criteria for precise tumour diagnoses [1]. One of the most challenging tasks in clinical oncology is to correctly classify and code clinical findings, using a multitude of available coding systems.

By far, the most important coding system for tumour staging is the Tumour-Node-Metastasis (TNM) classification [2] for malignant tumours, published by the Union for International Cancer Control (UICC)^1^. Besides a growing number of reliable biomarkers, TNM classification and staging are the most important information for the therapy planning for patients with colorectal cancer [3–5] and other solid tumours (e.g. cancer of the head and neck [6] or breast tumours [7]), except cancers of the central nervous system. In addition, the TNM classification system is important in cancer research for a correct description and classification of the anatomical extent of a given tumour. This is not only relevant for cancer epidemiology but also in fundamental tumour research (e.g. the dataset descriptions for researchers of the Surveillance, Epidemiology, and End Results Program (SEER) of the National Cancer Institute^2^ and predefined results using TNM stratified data^3^).

The TNM coding procedure requires advanced skills, encompassing both experience in tumour documentation and in-depth domain knowledge. The criteria for classification of the different primary tumour locations differ to the same extent as the underlying diseases. As a consequence, even expert coders and physicians for one organ system might encounter difficulties in the correct application or interpretation of TNM in a different organ system. Several combinations of tumour findings are difficult to encode due to ambiguous or overlapping criteria (non-disjoint definitions) or non-exhaustive definitions, which often result in cases where no TNM code or more than one TNM code is applicable to a given tumour state. A variety of problems with TNM coding has been described for different tumour locations. Main issues that arise in the practice of TNM coding derive from overly complex definitions of the underlying medical situation, which then result in interpretation problems even for experts [8–10]. The required in-depth knowledge of the domain, together with specific competences needed for TNM coding, result in poor coding completeness and quality, especially with the clinical staging in outpatients [11, 12]. Given the importance of TNM staging for the individual patient, deviation rates of about 20 % for clinical coding and 10 % for pathological coding can be interpreted as very high [13].

The complexity of TNM is mainly due to the development of the TNM classification as an evolutionary process [14], which has been constantly incorporating huge amount of new scientific insights in tumour prognosis and the dependency of therapeutic effects on tumour stage. Controlled by medical experts, TNM’s underlying structure has become more and more complex over the years. Experts in different fields of oncology have demanded a change in TNM maintenance, to address the increasing complexity, the detachment from clinical practice, and the resources needed for documentation [15, 16]. Therefore, standardisation of tumour classification and staging is an urgent requirement for improvement of tumour documentation in primary documentation, clinical studies and cancer registries [11, 17–20].

Despite its importance and formal precision, to the knowledge of the authors, no formal representation of the complete TNM is available so far. Formal, i.e. computable representations would have several advantages over TNM’s current publication as a textbook. An initial attempt to represent staging of lung tumours and glioma tumours was not continued [21, 22]. More recently, a description logics based (DL) approach was presented [23].

One of the major requirements a formal representation of TNM could satisfy is the automatic classification of instance data obtained from clinical databases or mined from textual reports [24–26]. Consecutively, instance data classification could inform higher order processes such as clinical documentation systems. Instance data on pathological or clinical conditions are collected during routine health care processes in pathology or other clinical information systems. Users could be supported by automatic encoding of instance data to TNM in real time or in spatially and temporally disseminated settings (e.g. in tumour documentation). For intelligent documentation systems in clinical oncology and pathology, a TNM ontology could be deployed as part of the knowledge base supporting the coding of tumour-related findings and the interpretation of TNM codes. In such systems a TNM ontology could enable automated reasoning based in description logics, which would timely detect logical inconsistencies and complexity related coding problems in databases and textual reports. In integrated clinical decisions support systems (DSS) TNM could be deployed to inform users about guideline-conformant treatment [27]. A further advantage of a formal approach would be the enhanced support for development and refinement of TNM. With a taxonomic backbone and axiomatic descriptions, the current complex natural language descriptions could be converted into computable structures. This would help decompose the descriptions into all their defining criteria, which in turn could facilitate the detection of coding errors, inconsistencies, and ambiguities in definitions [28, 29].

Description logics is the method of choice for a formalization of TNM [30]. Advanced retrieval and querying tools would be additional benefits that come with a logical representation following principles of Applied Ontology [31]. For these use cases, a formalised TNM version could constitute a unified source on which a variety of clinical documentation and analysis tools could be based. In addition, such a resource could be mapped to other DL-based clinical ontologies, especially to SNOMED CT.

With this work, we propose to close the gap of a missing formal representation by outlining and prototyping the TNM ontology (TNM-O). Following up on initial attempts in the breast cancer domain [32], the objectives of this work are (1) to present an ontological framework for the TNM classification system, (2) to implement a TNM ontology, describing colon and rectum tumours based on this framework, and (3) to evaluate this ontology using a tool for classifying pathology data.

### The TNM classification

The canonical description of the TNM classification based on the anatomic extent of disease (EOD) is published by the UICC and the AJCC [2, 33]. The UICC published the first edition of the TNM coding system in 1968. Since then, the system has undergone several revisions, with the 7^th^ edition published in 2009. The AJCC has recently announced the release of the 8^th^ edition of the TNM classification for the beginning of 2017^4^. The part of the new version for lung cancer is already in use with its important changes satisfying urgent medical requirements [34]. The objectives of the TNM coding system are six-fold. It supports treatment planning, prediction of outcomes (prognosis), evaluation of treatment results, exchange of information between different participants in health care processes, continuing research in malignant diseases, and cancer control [2, 14].

The *core* TNM classification uses three descriptors: T (tumour), N (metastasis in regional lymph nodes), and M (distant metastasis). The extent of the disease is indicated by integer values resp. character modifiers: TX (Tumour cannot be assessed), T0 (No evidence of primary tumour), T1-4 (increasing size or local extent), Tis (Carcinoma in situ); NX (Regional lymph nodes cannot be assessed), N0 (No regional lymph node metastasis), N1-3 (Increasing involvement of regional lymph nodes); M0 (No distant metastasis), M1 (Distant metastasis). For some entities further subdivisions of the categories are possible indicated by lower case characters (e.g. N2a and N2b).

The specific medical denotation for the different descriptors is dependent on the localisation of the tumour, designated by the ICD-O localisation code^5^. It is not possible to list all single regions addressed by the TNM classification here (for a current list see [2]). However, the TNM classification is not available for all body regions or systemic malignancies (e.g. C70-C72 Tumours of the Central Nervous System, C33 Trachea, C42, and C77 Tumours of haematopoietic and lymphoid tissues). For most of these malignancies the anatomical extent is either not determinable (systemic malignancies e.g. leukaemia) or the tumours have no metastasis (e.g. CNS tumours). The World Health Organisation (WHO) has published the 3*rd* edition of International Classification of Diseases for Oncology (ICD-O) in 2003. As an extension of the International Classification of Diseases (ICD-10) [35] for tumour diseases, the ICD-O is a dual classification system for the tumour morphology and the tumour localisation [36]. ICD-O is widely used in clinical medicine, tumour documentation, and research to encode tumour morphology and tumour localisation.

With an additional modifier, the TNM classification is divided into the pre-treatment *clinical* (indicated as cTNM) and post-surgical *pathological* (pTNM) classification. pTNM codes can only be assigned to the disease after pathological assessment following surgery and is the most important diagnostic item for following (adjuvant) radio- or chemotherapy or their combination. The results from the *clinical* assessment have to be accurately discerned from the *pathological* assessment due to their different meanings and evidence levels.

Besides the already complex semantics of the main numeric TNM codes, a series of additional symbols exists, which might have largely different meanings in the different tumour locations. Prefixes, suffixes, and certainty factors increase the confusion, e.g. for *carcinoma in situ* the suffix “is” has to be used (“Tis”). As TNM allows putting an “X” wherever the information about the clinical or pathological situation is incomplete or inaccurate, incomplete code assignments become widespread (e.g. MX for “no statement on metastases possible”). In this work only the classes with the descriptors T, N, and M with the modifiers c and p are represented (for a full list see Table 1).

**Table 1:** TNM classification descriptors and additional modifiers

Descriptor	Values	Meaning
T	0-4, is, X	Extent of the primary tumour
N	0-3, X	Extent of metastasis in regional lymph nodes
M	0-1	Existence of distant metastasis
Prefix to T, N, M	p, c	Clinical (pre-therapeutical) or pathological (post-surgical assessment)
Suffix to pNn	(mi)	Micrometastasis (< 0.2 cm)
Suffix to pNn	(sn)	Sentinel lymph node metastasis
Suffix to pN0 or pM0	(i+), (mol+)	Isolated tumour cells, positive findings
G	X, 1-4	Histopathological grading
Suffix to T	(m)	Multiple primary tumours at a single side
Prefix to c/ p	y	Assessment during multimodal therapy
Prefix to c/ p	r	Recurrent tumour
Prefix to c/ p	a	Assessment during autopsy
L	X, 0-1	Lymphatic invasion
V	X, 0-2	Venous invasion
Pn	X, 0-1	Perineural invasion
C	1-5	Validity of the assessment, can follow each of T, N, M
R	X, 0-2	Residual tumour

Depending on the organ of the primary tumour, T, N, and M values can be further subdivided into levels a-c, e.g. N1a-c, N2a-c, and M1a-b in colorectal tumours

pTNM codes are grouped into stages which are based on the prognosis of the patients. Stages are designated by the roman numerals I-IV and further subdivided into substages described by capital letters A-C. TNM staging has been subject to frequent changes during the history of the TNM classification, according to scientific and medical progress [34]. The mapping of the TNM classification for colon and rectum tumours to stages for version 7 is provided in [2, 4].

## Methods

TNM-O, the TNM ontology presented here, uses the Foundational Model of Anatomy [37] for anatomical entities, together with BioTopLite 2 (BTL2) as a domain top-level ontology [38, 39]. Tailored for the biomedical domain and based on description logics [30], BTL2 provides upper-level types both for general categories like *Material object*, *Process*, *Information object*, *Quality* etc., as well as constraints on all of them, using a set of sixteen canonical relations, partly derived from the OBO Relation Ontology (RO) [40]. They constrain each category by means of a set of general class axioms. BTL2 also contains other axioms such as relationship chains, existential and value restrictions. Thus, the building of domain ontologies under BTL2 heavily constrains the freedom of the ontology engineer, which is fully intended as it guarantees a higher predictability of the outcomes of the domain ontology production under BTL2.

The design of BTL2 is top-level agnostic and has been influenced both by the Basic Formal Ontology (BFO and BFO2) and the Descriptive Ontology for Linguistic and Social Engineering (DOLCE) which is discussed in more detail in [39]. BTL2 is especially appropriate as domain top-level for TNM-O because it provides a lean, yet exhaustive ontological framework for the representation of clinical documentation artefacts. Moreover, it is fully axiomatised using RO (see above) so that it is interoperable with other ontologies in the biomedical domain.

The development of TNM-O is an ongoing process. For this study, colorectal cancer was chosen as use case for several reasons. It is the third most common cancer worldwide and accounts for 9 % of all cancer incidence [41, 42], affecting more than one million humans in 2002. Treatment of cancer patients and research on causes of cancer are main goals of worldwide cancer control programs^6^. In prior work, the TNM classification for breast tumours (ICD-O C50) had been formally represented [32]. The selection of breast and colorectal tumours was motivated both by their paramount medical importance and their complexity in TNM, where both follow non-trivial medical classification principles, especially for the cN and pN classifications. Demonstrating the appropriateness and feasibility of TNM-O for these two tumour locations provides a good support for the general applicability of the approach.

The general rules of the TNM classification and the specific TNM classification for tumours of the colon and the rectum (ICD-O topography chapters C18 – C21, for ICD-O morphology codes see Table 2) were represented as described [2, 43].

**Table 2:** ICD-O 3 morphology codes for tumours of the colon and the rectum

Type	ICO-O 3 morphology
Adenocarcinoma	8140/3
Mucinous adenocarcinoma	8480/3
Signet-ring cell carcinoma	8490/3
Small cell carcinoma	8041/3
Squamous cell carcinoma	8070/3
Adenosquamous carcinoma	8560/3
Medullary carcinoma	8510/3
Undifferentiated carcinoma	8020/3

A classifying tool for individuals (instances) derived from pathology reports was developed employing the OWL API (version 4.0.1)^7^ and the HermIT DL reasoner (version 1.3.8)^8^. It classifies breast tumour and colorectal tumour data based on the corresponding TNM ontologies. It reads either tabular input data from files or processes data from manual entry via a graphical user interface.

The objective of TNM-O is not to re-design an existing tumour classification into a new system. At the current level of development, TNM-O is the result of an ontological analysis of what has been developed by the medical community over a long period, followed by its translation into a formal language, incorporating ontological principles, in order to improve the development, maintenance, and application of the TNM classification system.

In the following two sections, we describe (1) the TNM classification in detail as foundation of what has to be represented by TNM-O, (2) how the TNM classification artefacts are represented by information artefacts of TNM-O, (3) how these information artefacts are related to the actual tumour entities, and (4) how the patho-anatomical reality of tumour disease is constructed in terms of what is required for the TNM classification.

### Design of the TNM-O

The relation between the artefacts of the TNM classification and the actual tumour diseases is denotational: the T code denotes the extent (size, infiltration) of the primary tumour, the N code the extent of regional lymph node metastases, and the M code the existence of distant metastases. For TNM-O, we adopted an approach which is compliant with the Information Artefact Ontology from the OBO Foundry and recently published work on the *aboutness* relation [44, 45]. In TNM-O, coding artefacts of the TNM classification i.e. the classes of the classification are represented by subclasses of *btl2:InformationObject* as *RepresentationalArtefact*. Information reported on individual patients, e.g. as TNM-codes in patient records are thus individuals of these classes. Individuals from subclasses of *InformationObject* are related by **btl2:represents** to individuals of classes about the current disease state (*AnatomicalStructure*). The inverse relation is **btl2:isRepresentedBy** connects material or processual entities with the respective TNM-artefact.

As the TNM classification is compositional, the individual classes of the three descriptors can be *independently* combined to a joint code. Classes are only dependent on the location of the primary tumour and additional modifiers *c* or *p*: e.g. cN1 for colon cancer has a different meaning than cN1 for breast cancer, and cT1 has a different meaning than pT1 for all locations where these codes are available). This characteristic is conserved in TNM-O. The class *RepresentationalUnit* is a superclass of organ specific classes separated in a *clinical* and a *pathological* branch.

For representing anatomical structure, TNM-O uses content from the Foundational Model of Anatomy, restricted to cancer-related anatomy as referred to by the TNM classification. All primary tumours individuals and metastases are then related to individuals anatomical entities by the relation **btl2:locatedIn**, thus providing them with an exact topography and extent. The extent of primary tumours cannot only be described by their localisation (i.e. occupying space or infiltrating through layers of an organ) but can be further characterised by qualities, e.g. tumour size or infiltration patterns. These qualities are dependent on the localisation of the primary tumour and can substantially differ between them.

What makes a lymph node a *regional* lymph node depends on its proximity to a primary organ. An axillary lymph node is a regional lymph node of the breast gland but not of the colon. For all relevant organs, these regional lymph node groups are to be defined. Moreover, the formalisation of *infiltrated regional lymph nodes* depends on the aggregate of a localised primary tumour together with some metastasis in a regional lymph node of that organ in which the primary tumour is located. Thus, an infiltrated axillary lymph node is a regional lymph node metastasis for a breast tumour, but certainly not for a colon cancer. Distant metastases are, by definition, those located in a tumour aggregate that is not a regional lymph node of the primary tumour.

### Classification of pathology data

We computationally classified data describing the extent of 291 colorectal cancer specimens into TNM, documented at the Institute of Surgical Pathology, Medical Center – University of Freiburg using a pathology information system. This data were re-coded as RDF-OWL instance data and classified into classes of TNM-O by an application based on the OWL API using an OWL classifier^9^. Automatic classification was solely based on axioms defined in the colorectal TNM-O version 7 (TNM-O_colon_7.owl). The complete set of criteria is shown in Table 3.

**Table 3:** Criteria of TNM version 7 for colorectal cancers. All TNM codes can be inferred from this criteria. The exact wording of the textual definitions of the TNM in version 7 is diverging. Exact count of infiltrated organs in distant metastasis is omitted

Criterion	btl2 superclass	Value
Primary tumour extension	MaterialObject	Epithelium, Submucosa, Lamina propria, Subserosa, Adventitia, VisceralPeritoneum
Primary tumour growth pattern	Quality	Infiltrative, Confined
Primary tumour epistemology	Quality	NoAssessment, NoEvidence
Regional LN number	Quality	Cardinality1, Cardinality2or3, Cardinality4to6, Cardinality7orMore
Regional LN epistemology	Quality	NoAssessment, NoEvidence
Distant Mx location	MaterialObject	Peritoneum
Distant Mx/no. of organs	Quality	Cardinality1, Cardinality2orMore
Distant Mx epistemology	Quality	NoEvidence

http://cancerstaging.blogspot.de/2005/02/colon-and-rectum.html

For comparison of the ontology-based TNM classification with a manual expert TNM classification, the data were manually classified by a pathologist into TNM version 7.

## Results

TNM-O is designed as a modular system of independent ontologies under BTL2. For every organ or organ system based module of the TNM classification system, TNM-O provides a set of specific ontologies. The TNM connecting ontology serves as a hub to import BTL2 as well as the organ and organ system specific TNM ontologies (see Table 4). With the modular architecture only those modules are included that are needed by a tumour-specific application.

**Table 4:** Modular structure of TNM-O. Codes in clinical documentation and cancer registries follow TNM versions, because the meaning of codes and stages may change between versions. The modular structure is designed to include versions for every available TNM encoded entity (tumour location) so that the intended meaning is preserved according to the version used for coding

Name	Description
BTL2	Upper domain level ontology
TNM-O	TNM-O central connecting ontology
TNM-O_breast_7	TNM-O for breast cancer (TNM version 7) in: [32]
TNM-O_colorectal_6	TNM-O for colorectal cancer (TNM version 6)
TNM-O_colorectal_7	TNM-O for colorectal cancer (TNM version 7)

The hub TNM Ontology for all tumours can be downloaded from http://purl.org/tnmo/TNM-O.owl. The ontologies for breast tumours and colorectal tumours are named according to Table 4 and can be downloaded from the same site. They need to be loaded in the hub ontology.

Without inclusion of BTL2, the TNM hub ontology has the description logic expressivity of $\mathcal {A}LC$ (for a short introduction to the DL nomenclature see [46] section Description Logic Nomenclature). It consists of 79 axioms, 38 logical axioms, and 39 classes. It includes 35 subClassOf and one EquivalentTo axioms. Most of the classes are proxy classes to BTL2. Inclusion of BTL2 changes the DL expressivity to $\mathcal {S}RI$.

The TNM ontology for colorectal tumours has the description logic expressivity of $\mathcal {A}LC$. For TNM version 7.0 (version 6.0 in brackets), it consists of 366 (357) axioms, 198 (199) logical axioms, and 161 (149) classes. It includes 123 (160) subClassOf, 57 (18) EquivalentTo and 18 (18) DisjointClasses axioms.

### Representational units in the TNM-Ontology

The representation of the TNM system is decomposed into the representational units T, N, and M, together with the location of the primary tumour. Thus, for every existing code Tn, Nn, and Mn in combination with a specific organ there exists one *TNM-O:RepresentationalUnit* which is an *btl2:InformationObject*. E.g. every TNM code for colorectal cancer is represented by a separate class. Axioms using the relation **btl2:isRepresentedBy** introduce possible TNM values for subclasses of *PrimaryTumour* or *TumourAggregate*. This is done by connecting these values via the *universal* quantifier ONLY (role restriction). In all of these cases, the clause “or (not RepresentationalUnitInTNMClassification)” allows other values that are not TNM representational units. In the remaining text, the namespace of the TNM ontology is suppressed for clarity:

*TumourOfColonAndRectumWith7OrMoreMetastaticRegional- LymphNodes* subClassOf*TumourAggregate* and**btl2:isRepresentedBy** only (*ColonRectumTNM_pN2b* or *ColonRectumTNM_N2b* or (not *RepresentationalUnitInTNMClassification*))

### Representation of the primary tumour

The primary tumour is represented as *PrimaryTumour*, a subclass of *MalignantAnatomicalStructure*. The tumour characteristics relevant for the representational unit *T* of the TNM classification system are represented as location and qualities of *PrimaryTumour*. For colorectal tumours, the exact localization of the tumour in the gut wall, the quality of the tumour confinement with respect to neighbouring organs (confined or invasive), the quality of the assessment (no assessment, no evidence or carcinoma in situ), are important:

*InvasiveTumourOfSubmucosaOfColonAndRectum*EquivalentTo *ColonAndRectumTumour* and (**btl2:isBearerOf** some (*Confinement* and (**btl2:projectsOnto** some *Invasive*))) and (**btl2:isIncludedIn** some*SubmucosaOfLargeIntestine*)

The specific tumour defined as subclass of *PrimaryTumour* above is directly related to the corresponding representational unit as introduced in the section above.

*InvasiveTumourOfSubmucosaOfColonAndRectum*subClassOf**btl2:isRepresentedBy** some (*ColonRectumTNM_T1* or*ColonRectumTNM_pT1*) and**btl2:isRepresentedBy** only (*ColonRectumTNM_T1* or*ColonRectumTNM_pT1* or (not *RepresentationalUnitInTNMClassification*))

### Representation of regional lymph nodes

The most complex part of the TNM classification of many primary tumour locations is the interpretation of the axis *N*, which describes the extent of infiltration of regional lymph nodes by the primary tumour. The anatomy of lymph nodes draining the colon and rectum was modelled according to clinical anatomical conventions. Metastatic regional lymph nodes can exactly be located by the exact subclass of infiltrated regional lymph node:

*MetastaticLymphNodeOfColonAndRectumTumour*EquivalentTo *LymphNode* and (**btl2:hasPart** some*MetastasisOfColonAndRectumTumour*)

*MetastaticRegionalLymphNodeOfColonAndRectumTumour*EquivalentTo*MetastaticLymphNodeOfColonAndRectumTumour* and*ColonAndRectumRegionalLymphNode*

To define regional lymph node metastases of colorectal cancers, the aggregate of primary tumour and infiltrated lymph nodes around the colon and rectum (*TumourAggregate*) has to be considered as one (composite) entity. The representational unit *N* of the TNM classification of colorectal cancers depends on the count of metastatic regional lymph nodes and the presence of subserosal tumour deposits without regional lymph node metastases. The count of metastatic lymph nodes is represented by subclasses of *CardinalityValueRegion*:

*TumourOfColonAndRectumWith2or3MetastaticRegional- LymphNodes* EquivalentTo*TumourOfColonAndRectumWith1to3MetastaticRegional-* *LymphNodes* and (**btl2:isBearerOf** some (*Cardinality* and (**btl2:projectsOnto** some*Cardinality2or3*) and (**btl2:projectsOnto** only*Cardinality2or3*)))

### Representation of distant metastases

For the representational unit *M* of the TNM classification system the existence and number of distant metastases are evaluated. The definition of distant metastases excludes *regional* lymph nodes as their localisation:

*DistantMetastasisOfColonAndRectumTumour* EquivalentTo*MetastasisOfColonAndRectumTumour* and (not (**btl2:isIncludedIn** some*ColonAndRectumRegionalLymphNode*))

*TumourOfColonAndRectumWithDistantMetastasis*EquivalentTo*TumourOfColonAndRectumAggregate* and (**btl2:hasPart** some*DistantMetastasisOfColonAndRectumTumour*)

*TumourOfMammaryGlandWithDistantMetastasis*subClassOf (**btl2:isRepresentedBy** only (*MammaryGlandTNM_M1* or*MammaryGlandTNM_pM1* or (not *RepresentationalUnitInTNMClassification*))

### Classification of pathology data

**Fig. 1:**
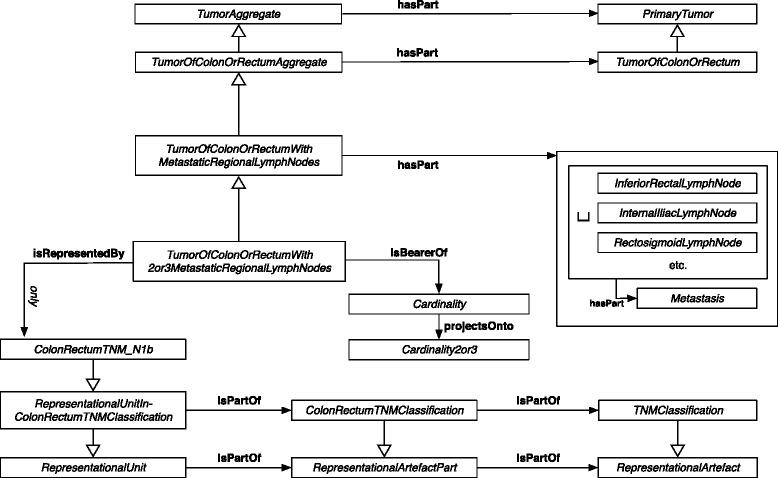
N1b representational unit of TNM-O for colorectal tumours. Graph of the patho-anatomical structures represented by an N1b representational unit of the TNM-O for colorectal tumours version 7 (TNM-O_colorectal_7.owl). T and M representational units are unspecified

**Fig. 2:**
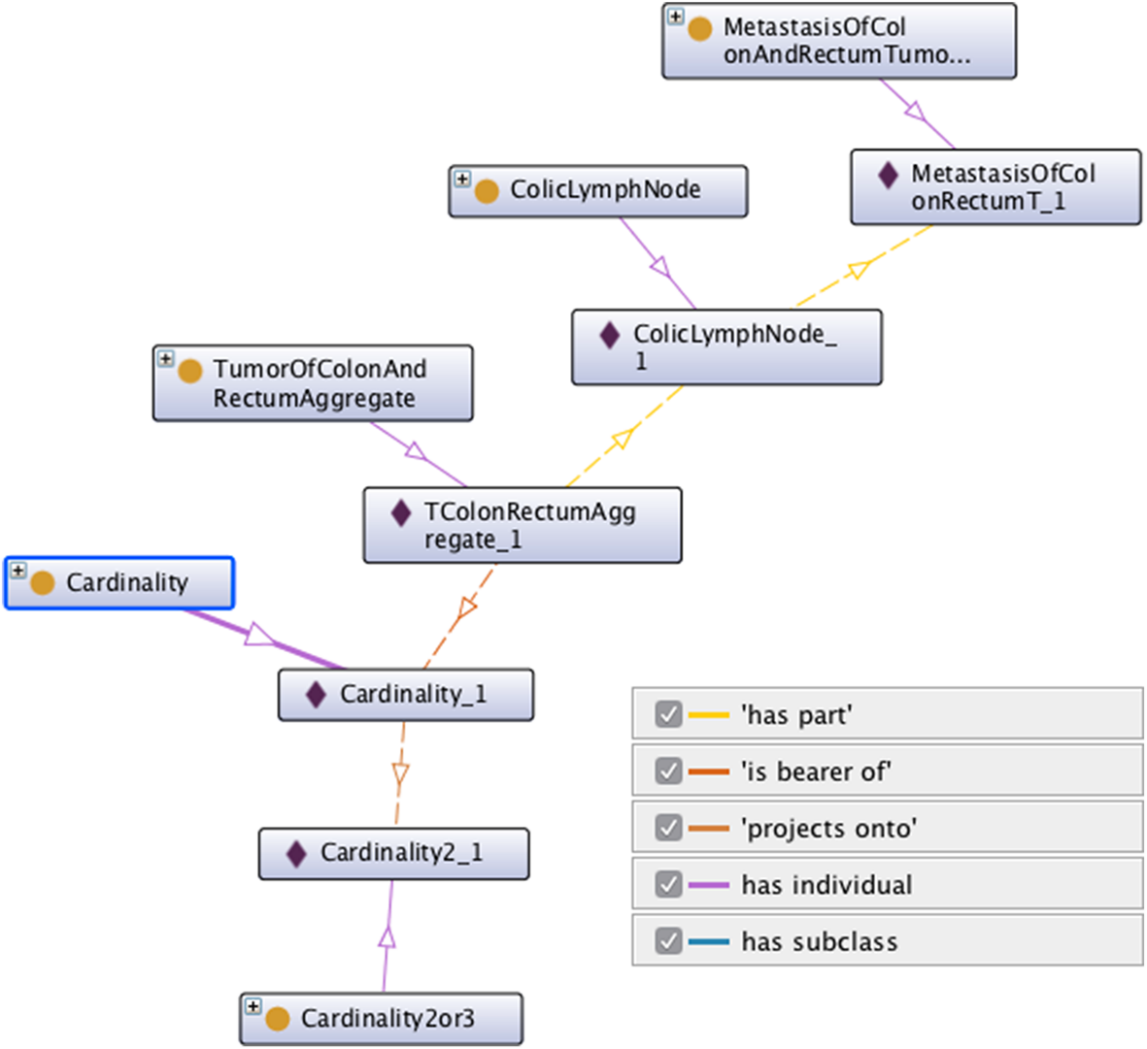
RDF-OWL instance of a tumour aggregate and corresponding OWL classes. Graph of an RDF instance of a tumour aggregate as created from tabular data according to TNM-O for colorectal tumours version 7 (TNM-O_colorectal_7.owl). RDF instances data are depicted with a purple diamond. RDF instance for T and M classification are omitted. Instances of this type are classified as TNM N1b

All instance data of 291 samples of colorectal cancer could be classified into classes of TNM-O on colorectal cancer. *A posteriori* comparison of the automatic classification results with a manual TNM coding based on the same findings from the pathology database by an experienced pathologist showed 100 % agreement. Table 5 shows 15 exemplary tabular instance data rows and the corresponding manual and automatic classification results. Figures 1 and 2 shows an example of an RDF-OWL instance which corresponds with rows 6 and 8 of Table 5. For clarity, the RDF example focuses on TNM N, other details on tumour invasion and distant metastasis were left out. All automatic classification results are based on TNM-O, TNM-O_colorectal_7 and RDF-OWL instance data.

**Table 5:** TNM relevant tabular data, manual expert TNM classification (subscript P), and ontology-based automatic TNM classification (subscript O)

Invasion of	rLN	tp rLN	TD/ Sat.	dMT	ip dMT	T_P_	N_P_	M_P_	T_O_	N_O_	M_O_
Subserosa	31	0	no	0	no	pT3	pN0	M0	pT3	pN0	M0
Muscular layer	13	0	no	0	no	pT2	pN0	M0	pT2	pN0	M0
Subserosa	19	0	no	0	no	pT3	pN0	M0	pT3	pN0	M0
Submucosa	18	0	no	0	no	pT1	pN0	M0	pT1	pN0	M0
Muscular layer	11	0	no	0	no	pT2	pN0	M0	pT2	pN0	M0
Visc. peritoneum	19	2	no	0	no	pT4a	pN1b	M0	pT4a	pN1b	M0
Subserosa	20	0	yes	0	no	pT3	pN1c	M0	pT3	pN1c	M0
Subserosa	14	2	no	0	no	pT3	pN1b	M0	pT3	pN1b	M0
Muscular layer	14	0	no	0	no	pT2	pN0	M0	pT2	pN0	M0
Subserosa	24	4	no	0	no	pT3	pN2a	M0	pT3	pN2a	M0
Other	16	6	no	0	no	pT4b	pN2a	M0	pT4b	pN2a	M0
Subserosa	17	0	no	0	no	pT3	pN0	M0	pT3	pN0	M0
Visc. peritoneum	40	29	no	0	no	pT4a	pN2b	M0	pT4a	pN2b	M0
Subserosa	15	0	no	0	no	pT3	pN0	M0	pT3	pN0	M0
Visc. peritoneum	24	15	no	1	no	pT4a	pN2b	M1a	pT4a	pN2b	M1a

rLN: Number of regional lymph nodes inspected; tp rLN: Number of tumour-positive regional lymph nodes, TD/ Sat.: Tumour deposits/ satellites; MT: Number of distant metastases; ip MT: Intra-peritoneal metastases

## Discussion

TNM is a globally accepted system to describe the anatomical extent of malignant tumours [2, 14]. Although TNM is of high importance for tumour staging, to the knowledge of the authors, there exists no comprehensive formal representation of TNM so far. With this work, the authors provide a first version of a TNM ontology (TNM-O) and a prototypical implementation of TNM for colorectal cancers. Further, this work shows that TNM-O classifies instance data.

Over time, TNM has developed into a coding system, which had to accommodate both the pragmatics of coding and representational accuracy. The literature on ambiguities and difficulties of TNM in practice is abundant. The discussion of TNM for breast tumours illustrates the dilemma of its maintainers [8, 47, 48]. They had to account for the rapid progression of scientific knowledge on tumours and to keep it usable at the same time: new versions of TNM are already outdated when compared with new scientific insights. On the other hand, TNM has become increasingly complex, with a negative impact on its usability by both expert and non-expert documentation staff and physicians.

Encoding clinical conditions using TNM as well as the selection of the right treatment according to TNM codes is daily routine in oncology. In order to assist in these difficult and time consuming decision processes, several systems have been proposed, usually based on text extraction from pathology reports and machine learning algorithms [24–26]. The accuracy of these approaches was relatively low [24]. Here, we present an ontology, which classifies instance data with 100 % accuracy in an experimental setting based on structured data. We hypothesise that DL based classification using TNM-O could also improve the results from automated information extraction from unstructured data as done in the above mentioned approaches. Such systems could also be made available in intelligent documentation systems in the form of embedded decision support systems, which could help to choose the right codes for a clinical condition and/ or the right guideline compliant treatment for a given code (describing a clinical condition). Furthermore, we think that with an ontology the curation of the TNM itself could be improved. Based on a taxonomic and axiomatic description, the detection of coding errors, inconsistencies, and ambiguities in definitions could be facilitated [28, 29]. A formal description logic based axiomatisation allows the use of specific reasoning tools to check for inconsistencies during the ontology engineering process, which would indicate conflicting axioms. Redundancies or wrong hierarchical dependencies is detected by checking the inferred class hierarchy after DL classification.

This study is limited as far as we provide here a *first version* of the TNM Ontology (TNM-O), limited to mammary gland [32] and colorectal tumours. As these two tumour entities are the most complex and best represented ones in TNM, the current version is already sufficiently complete and stable to be used as a blueprint for TNM-O extensions to other organ systems.

Due to the nature of the domain and the rich top-level ontology employed, the computational resources needed to classify the ontology are considerable. In order to alleviate performance issues, TNM-O will be provided as modules for different organ systems. Thus, the users can import only the modules of interest into their application context.

Future research should evaluate the presented prototype ontology (i) by implementing further tumour locations, and (ii) by systematic application in clinical classification and retrieval scenarios. We will provide the formalization of TNM for other primary tumour locations in a modular way, so that users can select which part of the TNM-O they would like to use. In this way, we hope to reduce the computational resources already needed to a minimum.

## Conclusion

We presented a first version of an ontology (TNM-O) that represents the TNM tumour classification system. The present work demonstrates its representational power and completeness as well as its applicability for classification of instance data. This work provides a foundation for an exhaustive TNM ontology.

## Endnotes

^1^ http://www.uicc.org

^2^ http://seer.cancer.gov/seerstat/databases/ssf/

^3^ http://seer.cancer.gov/csr/1975_2013/sections.html

^4^ https://cancerstaging.org/About/news/Pages/8th-Edition-Publication-Date-Announced.aspx

^5^ http://codes.iarc.fr/usingicdo.php

^6^ http://www.who.int/cancer/modules/en/

^7^ http://owlapi.sourceforge.net/

^8^ http://hermit-reasoner.com/

^9^ http://owlapi.sourceforge.net/
